# Motivation, Teaching Satisfaction, Loneliness and Affect in Spanish University Teachers: An Analysis of Motivational Profiles and of Differences by Age, Gender, Place of Residence and Field of Knowledge

**DOI:** 10.62641/aep.2405

**Published:** 2026-10-15

**Authors:** Víctor Renobell-Santarén, Sílvia Fuentes-de-Frutos

**Affiliations:** ^1^Faculty of Social Sciences, International University of La Rioja (UNIR), 26006 Logroño, La Rioja, Spain; ^2^Faculty of Education, International University of La Rioja (UNIR), 26006 Logroño, La Rioja, Spain

**Keywords:** teaching motivation, life satisfaction, loneliness, affect, university teachers, occupational mental health

## Abstract

**Background:**

Teacher motivation is a protective factor for psychological well-being and a recognised predictor of teaching quality, and its deterioration has been linked to anxious-depressive symptoms, emotional exhaustion and occupational loneliness. This study analysed the relationships among motivation, life satisfaction, loneliness and positive and negative affect in Spanish university teachers, identified latent motivational profiles through cluster analysis, and examined differences by age, gender, place of residence and field of knowledge.

**Methods:**

A cross-sectional online survey was conducted between February and June 2024 with 503 Spanish university teachers (mean age = 43.70 years; standard deviation (SD) = 9.80; 58.8% women), a sample size confirmed as sufficient by an a priori power analysis (G*Power 3.1). Participants completed a sociodemographic questionnaire, the Multidimensional Work Motivation Scale (R-MAWS), the Satisfaction With Life Scale (SWLS), the three-item University of California, Los Angeles Loneliness Scale and the Positive and Negative Affect Schedule. Analyses included descriptive statistics, Pearson correlations, *t*-tests, one-way analysis of variance with Bonferroni post hoc tests, k-means cluster analysis, hierarchical regression and a mediation model with 5000 bootstrap resamples (two-sided tests; α = 0.05). Common-method bias was assessed with Harman's single-factor test, and the cluster solution was validated through silhouette analysis and split-half replication.

**Results:**

Autonomous motivation and life satisfaction were robust positive predictors of positive affect (β = 0.48 and β = 0.29; both *p* < 0.001) and were associated with lower loneliness. Three profiles emerged: autonomous-flourishing (31.8%), ambivalent-intermediate (30.6%) and demotivated-vulnerable (37.6%). Significant differences appeared by gender in negative affect (*t*[501] = 5.65; *p* < 0.001; d = 0.51), by place of residence in loneliness (*t*[501] = –6.99; *p* < 0.001; d = –0.62) and by field of knowledge in life satisfaction (F[2, 500] = 8.75; *p* < 0.001; η^2^*p* = 0.03).

**Conclusions:**

Autonomous motivation acts as a protective factor for the occupational mental health of university teachers. Interventions should be sensitive to sociodemographic and territorial differences, and these cross-sectional associations require longitudinal confirmation.

## Introduction

University teaching takes place in an increasingly demanding work environment. This environment is marked by pressure for research productivity, by the precarious contracts of part of the academic workforce, and by the intensification of administrative and teaching demands. Against this backdrop, the study of teacher motivation and its links with psychological well-being has gained particular relevance within occupational mental health research. Contemporary psychiatry regards this field as central to preventing mood and anxiety disorders in the working population [1]. From the perspective of the Job Demands-Resources model, a sustained imbalance between occupational demands and available resources constitutes a proximal antecedent of emotional exhaustion and disengagement among teaching staff [2].

Within the framework of Self-Determination Theory, motivation is not a unitary phenomenon. Rather, it forms a continuum that ranges from amotivation to intrinsic motivation, passing through various forms of extrinsic regulation [2, 3, 4]. Autonomous motivation (in which the person perceives the activity as freely chosen and consistent with their values) is consistently associated with higher levels of satisfaction, positive affect and commitment. Controlled motivation and amotivation, by contrast, are linked to emotional distress, exhaustion and a greater risk of anxious-depressive symptoms [5, 6, 7]. The satisfaction of the basic psychological needs for autonomy, competence and relatedness is a robust protective mechanism against burnout across educational levels [8, 9]. Meta-analytic evidence in educational settings corroborates that need-supportive environments strengthen autonomous forms of regulation and adaptive outcomes [10], and autonomous motivation for teaching has been shown to foster self-determined learning among students [11].

Life satisfaction, understood as the cognitive component of subjective well-being, and affect, in its positive and negative dimensions, have been widely used as indicators of psychological well-being in occupational samples [12, 13]. In parallel, loneliness (defined as the perceived discrepancy between desired and actual social relationships) is recognised as a risk factor for mental and physical health. It has attracted growing attention in workplace settings following recent changes in the way work is organised [14, 15, 16]. Recent occupational evidence confirms that workplace loneliness is a prevalent and consequential phenomenon: a mixed-method systematic review and meta-analysis established its association with depression, anxiety and absenteeism [17], a six-month prospective study showed that it predicts subsequent job turnover [18], and an integrative review positioned loneliness as a central concern for contemporary organisational and occupational health research [19]. Psychiatric literature links loneliness to an increased risk of depression, cognitive decline and all-cause mortality, which justifies its inclusion as a core variable in the study of teacher well-being [20, 21]. The theoretical and empirical review by Hawkley and Cacioppo details the physiological and cognitive mechanisms through which perceived loneliness erodes health over time [22], while eudaimonic models locate these processes within a broader architecture of psychological well-being [23].

Prior evidence further establishes that these variables are unevenly distributed across sociodemographic characteristics. Gender differences have been reported in the experience of negative affect and loneliness, together with variations linked to age and to career stage [24, 25, 26]. Less attention has been paid to the effect of place of residence (which shapes access to social networks and services) and of field of knowledge. Yet disciplinary cultures (for instance, science, technology, engineering and mathematics (STEM) fields versus the health sciences or the social sciences and humanities) give rise to markedly different work settings, incentive systems and relational dynamics [27]. Consistent with this evidence, previous research on Spanish teachers documented sex-based differences in motivation, teaching satisfaction, loneliness and affect during the pandemic period [28].

A complementary approach to variable-by-variable analysis is the identification of motivational profiles through person-centred techniques, such as cluster analysis. This approach identifies subgroups of teachers who share a specific configuration of motivation and well-being, and it is especially useful for guiding tailored interventions [29, 30]. Previous studies of Spanish teachers have documented motivational profiles associated with different levels of satisfaction, loneliness and affect. Some findings, however, are counter-intuitive (for example, the co-occurrence of high motivation and high loneliness during periods of remote teaching), which calls for replication under conditions of normalised face-to-face activity [29].

A specific theoretical rationale underpins the expected mediating role of life satisfaction. Within Self-Determination Theory, autonomous motivation fosters the satisfaction of the basic psychological needs and a sense of congruence between one’s activity and one’s values [4, 5]. This congruence feeds the cognitive appraisal of life as a whole, which is precisely what the Satisfaction With Life Scale (SWLS) captures as the evaluative component of subjective well-being [12, 31]. Because global life evaluations operate as a relatively stable cognitive frame through which momentary experiences are interpreted, a teacher who appraises their life favourably translates autonomously regulated engagement into frequent positive affect more readily. Life satisfaction therefore constitutes a plausible cognitive pathway linking autonomous motivation to positive affect, a sequence that the present study tests empirically [13, 32].

Despite the relevance of the topic, few studies have jointly examined motivation, satisfaction, loneliness and affect in Spanish university teachers while also adopting a profile-based approach and systematically assessing differences by age, gender, residence and field of knowledge. The present study fills this gap and provides novel evidence on the territorial and disciplinary patterning of teacher well-being.

### Aims and Hypotheses

The general aim was to analyse the relationships among motivation, life satisfaction, loneliness and affect in Spanish university teachers, to identify motivational profiles, and to examine differences in these variables by age, gender, place of residence and field of knowledge. The following hypotheses were proposed: (H1) autonomous motivation and life satisfaction will be positively associated with positive affect and negatively associated with loneliness and negative affect; (H2) significant gender differences will emerge, with higher levels of negative affect and loneliness among women; (H3) differences by age, residence and field of knowledge will be observed in the study variables; (H4) distinct motivational profiles will be identifiable in their well-being patterns; and (H5) life satisfaction will mediate (statistical mediation) the relationship between autonomous motivation and positive affect.

## Materials and Methods

### Design and Participants

An observational, cross-sectional survey study was carried out. The sample comprised 503 Spanish university teachers, recruited through non-probability convenience and snowball sampling. Recruitment was conducted between February and June 2024 through three complementary channels: the institutional distribution lists of several Spanish public and private universities, the mailing lists of national academic and professional associations, and targeted dissemination on professional social-media networks (LinkedIn and X). The inclusion criteria were: (a) being engaged in teaching at a Spanish university at the time of the study, and (b) providing informed consent. Incomplete questionnaires were excluded, as were those with inconsistent response patterns (for example, uniform responding on reverse-worded items). Fig. 1 summarises the participant flow in accordance with the Strengthening the reporting of observational studies in epidemiology (STROBE) recommendations: of the initial respondents who accessed the questionnaire, those who did not provide consent were not allowed to proceed, and questionnaires that were incomplete or showed inconsistent or careless responding were removed, which yielded the final analytic sample of 503 participants. The mean age was 43.70 years (standard deviation (SD) = 9.80; range 26–64). The full sociodemographic distribution is shown in Table 1.

**Fig. 1.**
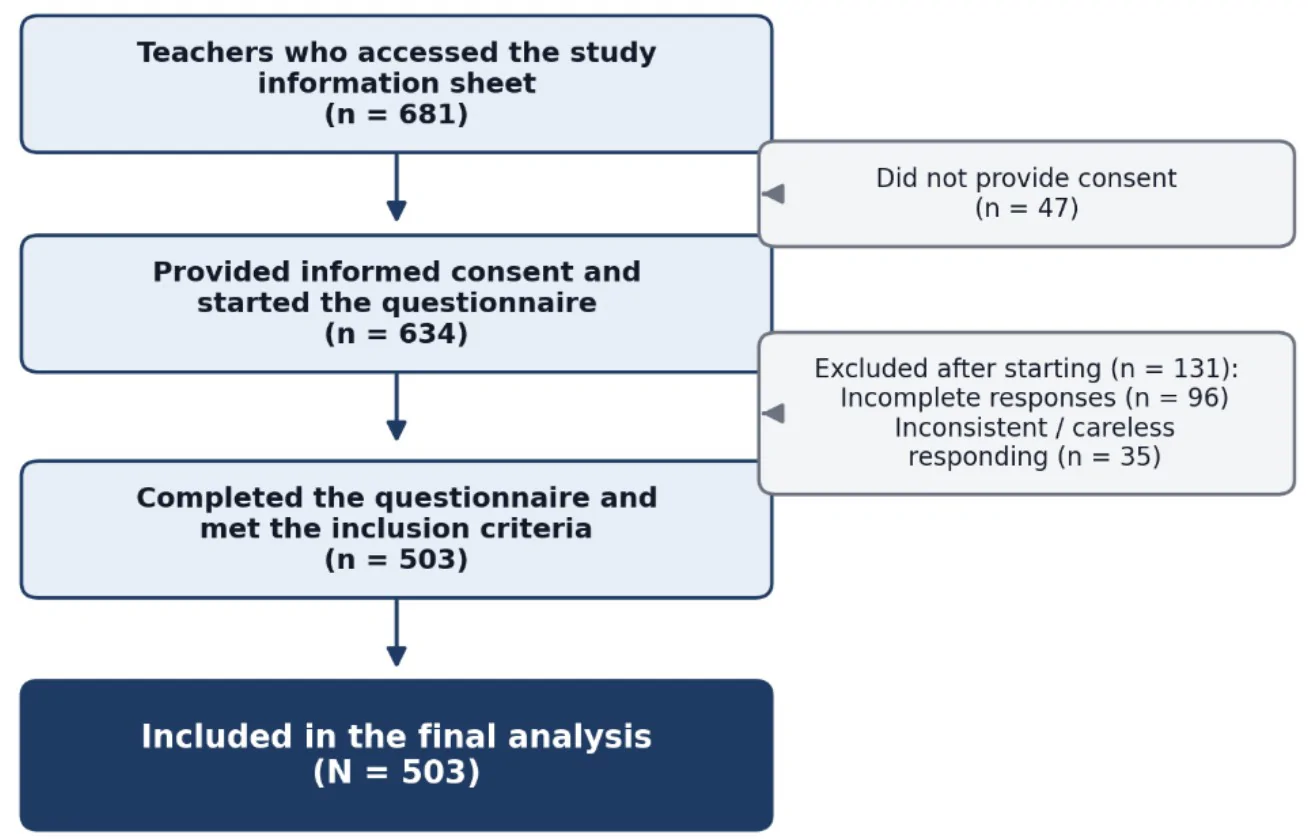
**Participant flow diagram**. Participant flow diagram. (STROBE format), showing the number of individuals who accessed the questionnaire, those excluded for absence of consent, incomplete responses or inconsistent responding, and the final analytic sample of 503 participants.

To contextualise the representativeness of the sample, its composition was compared with the official figures of the Spanish university system. According to the Ministry of Science, Innovation and Universities, women represented 44.6% of all teaching and research staff in 2023–2024, and the mean age of this workforce was 49.5 years [33]. The present sample therefore over-represents women (58.8%) and younger teachers (mean age = 43.70 years), a pattern that is common in voluntary online surveys and that is acknowledged as a limitation in Section Limitations. No post-stratification weighting was applied, so the descriptive proportions are sample-specific rather than population estimates.

### Instruments

All instruments used are freely available, have validated Spanish versions and are widely used in the prior literature.

#### Ad Hoc Sociodemographic Questionnaire

This recorded age, gender, place of residence, field of knowledge, professional category, years of teaching experience and type of contract.

#### Multidimensional Work Motivation Scale (R-MAWS)

This assesses work motivation according to Self-Determination Theory, distinguishing amotivation, external, introjected and identified regulation, and intrinsic motivation, which can be grouped into autonomous and controlled motivation indices [3]. The scale comprises 19 items rated from 1 (not at all) to 7 (completely); subscale scores were computed as item means and therefore range from 1 to 7. In the present sample, internal consistency was α = 0.89 (autonomous) and α = 0.81 (controlled).

#### SWLS

This comprises five items with a 7-point response format and measures the cognitive component of subjective well-being [12], yielding total scores from 5 to 35 and mean scores from 1 to 7. The Spanish adaptation validated in Spanish-speaking populations was used [34]. Reliability was α = 0.88.

#### UCLA Loneliness Scale (Three-Item Short Version)

This assesses perceived loneliness (lack of companionship, feeling left out and feeling isolated) with a 3-point response format (1 = hardly ever to 3 = often), so that total scores range from 3 to 9. It is widely used in population studies because of its brevity and sound psychometric properties [14]. Reliability was α = 0.82.

#### Positive and Negative Affect Schedule (PANAS)

This comprises 20 items (10 for positive affect and 10 for negative affect) with a 5-point response format [13] (1 = very slightly or not at all to 5 = extremely); each subscale score is the mean of its 10 items and ranges from 1 to 5. The Spanish factorial validation was used [35]. Reliability was α = 0.90 (positive affect) and α = 0.87 (negative affect).

### Procedure and Ethical Considerations

The questionnaire was administered online, between February and June 2024, through a survey platform hosted on servers with end-to-end encryption (HTTPS/TLS 1.3 protocol) and compliant with Regulation (EU) 2016/679 (GDPR) and Spanish Organic Law 3/2018 on the Protection of Personal Data and the Guarantee of Digital Rights. Recruitment was carried out through institutional mailing lists and professional networks. Participation was voluntary, anonymous and unpaid.

Before starting the questionnaire, participants read the study information sheet and gave their informed consent electronically by ticking an acceptance box, which was required in order to access the items. No directly identifying data (name, IP address or email) were collected, so that the resulting dataset was fully anonymous. The information was stored in encrypted form, with access restricted to the principal investigator through two-factor authentication. The data will be retained for a maximum of five years in accordance with institutional regulations. The study was conducted in accordance with the principles of the Declaration of Helsinki and was approved by the Research Ethics Committee of the International University of La Rioja (Exp. 107-2026).

### Data Analysis

Data were analysed using IBM SPSS Version 25 (IBM Corp., Armonk, NY, USA) and the PROCESS macro for IBM SPSS Statistics, Version 4.2 (Hayes, Columbus, OH, USA). Descriptive statistics were computed (means, standard deviations, skewness and kurtosis). Normality was assessed through skewness and kurtosis indices, with values between –2 and +2 deemed acceptable. Comparisons between two groups (gender, residence) were conducted with the independent-samples Student *t*-test, with effect sizes reported as Cohen’s d. Comparisons among more than two groups (age, field of knowledge) were carried out with one-way analysis of variance (ANOVA), using Bonferroni post hoc tests and partial eta squared ($\eta^{2}$*p*) as the effect-size statistic. Relationships among variables were examined using Pearson correlations.

To identify motivational profiles, a k-means cluster analysis was performed on the standardised scores of the six study variables. K-means was selected because the study variables were continuous, the expected profiles were non-overlapping, and the technique is robust and widely used for motivational profiling in educational samples [29, 30]. Two-, three- and four-cluster solutions were compared, and the three-cluster solution was retained on the basis of the elbow criterion, the average silhouette width and theoretical interpretability. The stability of the solution was confirmed through split-half cross-validation, in which the cluster structure was re-estimated in two random subsamples and yielded a consistent configuration [36]. The association between cluster membership and the sociodemographic variables was examined using chi-square tests, and pairwise differences among clusters were tested with Bonferroni-corrected post hoc comparisons. In addition, hierarchical multiple regression models were fitted to predict positive affect, and a mediation model was tested with 5000 bootstrap resamples (PROCESS model 4). The mediation analysis followed the contemporary statistical framework for indirect effects based on bias-corrected bootstrap confidence intervals [37]. Age was analysed both as a continuous, mean-centred predictor in the regression models and as a three-level categorical factor in the analyses of variance (Table 4); the two operationalisations yielded convergent conclusions. The significance level was set at *p* < 0.05. All statistical tests were two-sided. An a priori power analysis (G*Power 3.1.9.7, Heinrich Heine University Düsseldorf, Düsseldorf, Germany) indicated that, for a multiple regression with seven predictors, a medium effect size (f^2^ = 0.15), α = 0.05 and power = 0.80, the minimum required sample was 103 participants; the achieved sample of 503 therefore provided ample power, sufficient to detect even small effects (f^2^ = 0.02, requiring 395 participants). Common-method bias was evaluated with Harman’s single-factor test, submitting all items to an unrotated exploratory factor analysis, with a single factor accounting for more than 50% of the variance taken as the threshold of concern [38].

## Results

### Descriptive and Reliability Analyses

Table 1 presents the sociodemographic characteristics of the sample, and Table 2 the descriptive statistics and reliability coefficients. All Cronbach’s α coefficients exceeded 0.80, and McDonald’s ω values exceeded > 0.82, indicating adequate reliability. McDonald’s ω was reported as a complementary reliability index because, unlike coefficient α, it does not assume tau-equivalence of the items [38]. Skewness and kurtosis indices fell within the recommended range, supporting the assumption of univariate normality. Harman’s single-factor test supported the absence of a dominant common-method factor, since the first unrotated factor accounted for less than half of the total variance, below the conventional 50% threshold [39].

**Table 1.** **Sociodemographic characteristics of the sample (N = 503)**.

Variable	Category	n	%
Gender	Women	296	58.8
	Men	207	41.2
Age group	≤35 years	120	23.9
	36–50 years	240	47.7
	>50 years	143	28.4
Residence	Large city (≥ 250,000 inhab.)	267	53.1
	Rest of territory	236	46.9
Field of knowledge	STEM	176	35.0
	Health sciences	101	20.1
	Other (Social Sciences, Law and Humanities)	226	44.9

Note: The categories of each variable sum to the total sample. STEM, science, technology, engineering and mathematics.

**Table 2.** **Descriptive statistics and reliability of the scales (N = 503)**.

Variable	M	SD	Skewness	Kurtosis	α	ω
Autonomous motivation	5.53	0.84	–0.27	–0.31	0.89	0.90
Controlled motivation	3.63	1.00	–0.05	–0.21	0.81	0.82
Life satisfaction (SWLS)	4.20	1.04	0.12	0.00	0.88	0.89
Loneliness (UCLA-3)	2.06	0.47	–0.14	–0.55	0.82	0.83
Positive affect (PANAS-PA)	3.51	0.73	–0.15	–0.31	0.90	0.91
Negative affect (PANAS-NA)	2.52	0.57	–0.13	0.10	0.87	0.88

Note: M, mean; SD, standard deviation; α, Cronbach’s α; ω, McDonald’s ω. Motivation and satisfaction on a 1–7 scale; loneliness on a 1–3 scale; affect on a 1–5 scale. PANAS, Positive and Negative Affect Schedule; SWLS, Satisfaction With Life Scale; UCLA, University of California, Los Angeles Loneliness Scale.

### Correlations

Table 3 shows the Pearson correlations among the variables. Autonomous motivation correlated positively with life satisfaction (r = 0.40; *p* < 0.001) and positive affect (r = 0.62; *p* < 0.001), and negatively with loneliness (r = –0.39; *p* < 0.001) and negative affect (r = –0.29; *p* < 0.001). Loneliness, in turn, was positively associated with negative affect (r = 0.30; *p* < 0.001) and negatively with life satisfaction (r = –0.24; *p* < 0.001). This pattern is displayed in the heat map in Fig. 2.

**Table 3.** **Pearson correlation matrix among the study variables (N = 503)**.

Variable	1	2	3	4	5	6
1. Autonomous motivation	-					
2. Controlled motivation	–0.11*	-				
3. Life satisfaction	0.40***	–0.28**	-			
4. Loneliness	–0.39***	0.29**	–0.24***	-		
5. Positive affect	0.62***	–0.16**	0.50**	–0.25**	-	
6. Negative affect	–0.29***	0.13**	–0.12**	0.30***	–0.21**	-

Note: *p < 0.05; **p < 0.01; ***p < 0.001.

**Fig. 2.**
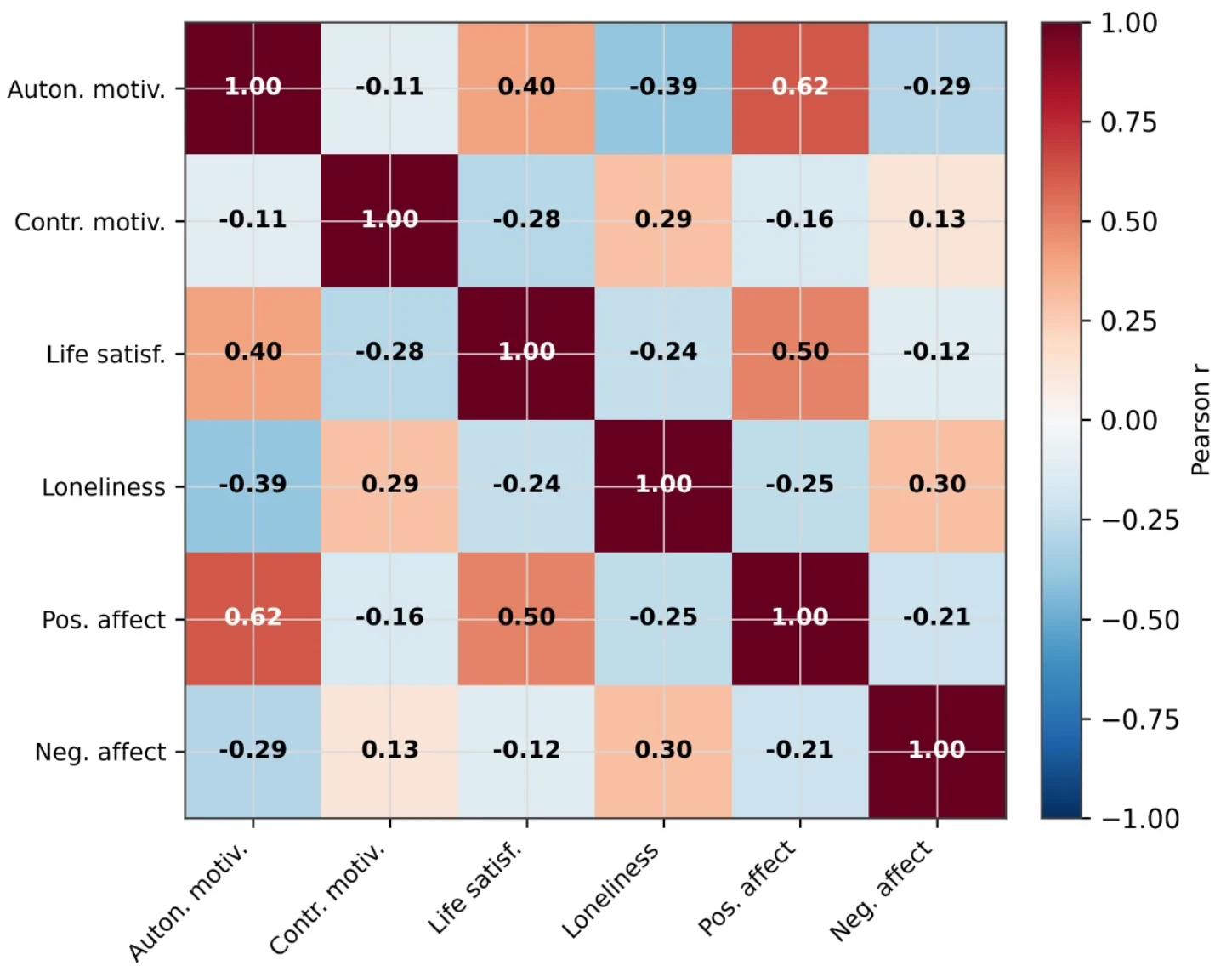
**Heat map of the Pearson correlation matrix among the study variables**.

### Group Differences

Throughout this section, interpretation is anchored on effect sizes rather than statistical significance alone, following Cohen’s conventions for small (d ∼ 0.20; $\eta^{2}$*p* ∼ 0.01), medium (d ∼ 0.50; $\eta^{2}$*p* ∼ 0.06) and large (d ∼ 0.80; $\eta^{2}$*p* ∼ 0.14) effects [40].

### By Gender

The independent-samples *t*-tests compared women and men on all six study variables (Table 4 and Fig. 3). Significant differences emerged in negative affect (*t*[501] = 5.65; *p* < 0.001; d = 0.51) and loneliness (*t*[501] = 2.57; *p* = 0.011; d = 0.23), with higher values among women. The remaining four comparisons were non-significant and are now plotted alongside the significant ones: autonomous motivation (*t*[501] = –0.91; *p* = 0.362; d = 0.08), controlled motivation (t[501] = 0.77; *p* = 0.441; d = 0.07), life satisfaction (*t*[501] = –0.74; *p* = 0.459; d = 0.07) and positive affect (*t*[501] = –1.05; *p* = 0.294; d = 0.10). Thus the gender effect was confined to the negative affective pole, with a medium effect for negative affect and a small effect for loneliness.

**Table 4.** **Mean comparisons of the six study variables by gender, age group, place of residence and field of knowledge (N = 503)**.

Variable (grouping factor)	Groups: M (SD)	*t*/F	*p*	d/$\eta^{2}$*p*
Autonomous motivation (gender)	W 5.50 (0.85)/M 5.57 (0.83)	*t* = –0.91	0.362	d = 0.08
Controlled motivation (gender)	W 3.66 (0.99)/M 3.59 (1.01)	*t* = 0.77	0.441	d = 0.07
Life satisfaction (gender)	W 4.17 (1.05)/M 4.24 (1.02)	*t* = –0.74	0.459	d = 0.07
Loneliness (gender)	W 2.10 (0.46)/M 1.99 (0.49)	*t* = 2.57	0.011	d = 0.23
Positive affect (gender)	W 3.48 (0.74)/M 3.55 (0.72)	*t* = –1.05	0.294	d = 0.10
Negative affect (gender)	W 2.64 (0.54)/M 2.36 (0.57)	*t* = 5.65	<0.001	d = 0.51
Autonomous motivation (age)	5.16 (0.90)/5.54 (0.82)/5.82 (0.78)	F = 21.91	<0.001	$\eta^{2}$*p* = 0.08
Controlled motivation (age)	3.95 (1.02)/3.66 (0.99)/3.28 (0.95)	F = 16.11	<0.001	$\eta^{2}$*p* = 0.06
Life satisfaction (age)	4.14 (1.06)/4.20 (1.04)/4.25 (1.02)	F = 0.72	0.487	$\eta^{2}$*p* = 0.003
Loneliness (age)	2.16 (0.46)/2.06 (0.47)/1.96 (0.45)	F = 7.78	<0.001	$\eta^{2}$*p* = 0.03
Positive affect (age)	3.22 (0.74)/3.53 (0.72)/3.73 (0.70)	F = 16.68	<0.001	$\eta^{2}$*p* = 0.06
Negative affect (age)	2.57 (0.58)/2.52 (0.57)/2.47 (0.56)	F = 1.05	0.351	$\eta^{2}$*p* = 0.004
Autonomous motivation (residence)	City 5.55 (0.83)/Rest 5.51 (0.85)	*t* = 0.53	0.594	d = 0.05
Controlled motivation (residence)	City 3.61 (1.01)/Rest 3.65 (0.99)	*t* = –0.45	0.653	d = 0.04
Life satisfaction (residence)	City 4.24 (1.02)/Rest 4.15 (1.06)	*t* = 0.97	0.331	d = 0.09
Loneliness (residence)	City 1.92 (0.44)/Rest 2.21 (0.46)	*t* = –6.99	<0.001	d = –0.62
Positive affect (residence)	City 3.54 (0.72)/Rest 3.48 (0.74)	*t* = 0.93	0.354	d = 0.08
Negative affect (residence)	City 2.47 (0.56)/Rest 2.58 (0.58)	*t* = –1.84	0.066	d = –0.19
Autonomous motivation (field)	STEM 5.50 (0.71)/HS 5.58 (0.64)/Other 5.53 (0.80)	F = 0.32	0.727	$\eta^{2}$*p* = 0.001
Controlled motivation (field)	STEM 3.68 (0.93)/HS 3.57 (0.89)/Other 3.62 (0.96)	F = 0.45	0.639	$\eta^{2}$*p* = 0.002
Life satisfaction (field)	STEM 3.94 (0.93)/HS 4.36 (0.99)/Other 4.33 (1.02)	F = 8.75	<0.001	$\eta^{2}$*p* = 0.03
Loneliness (field)	STEM 2.08 (0.51)/HS 2.03 (0.44)/Other 2.05 (0.42)	F = 0.51	0.598	$\eta^{2}$*p* = 0.002
Positive affect (field)	STEM 3.40 (0.81)/HS 3.70 (0.77)/Other 3.51 (0.73)	F = 5.44	0.005	$\eta^{2}$*p* = 0.02
Negative affect (field)	STEM 2.55 (0.53)/HS 2.48 (0.59)/Other 2.51 (0.50)	F = 0.68	0.510	$\eta^{2}$*p* = 0.003

Note: W, women; M, men; HS, health sciences; STEM, science, technology, engineering and mathematics; SD, standard deviation. Age groups: ≤35/36–50/>50 years, in that order. d, Cohen’s d; $\eta^{2}$*p*, partial eta squared. All age and field contrasts have df = (2, 500); gender and residence contrasts have df = 501.

**Fig. 3.**
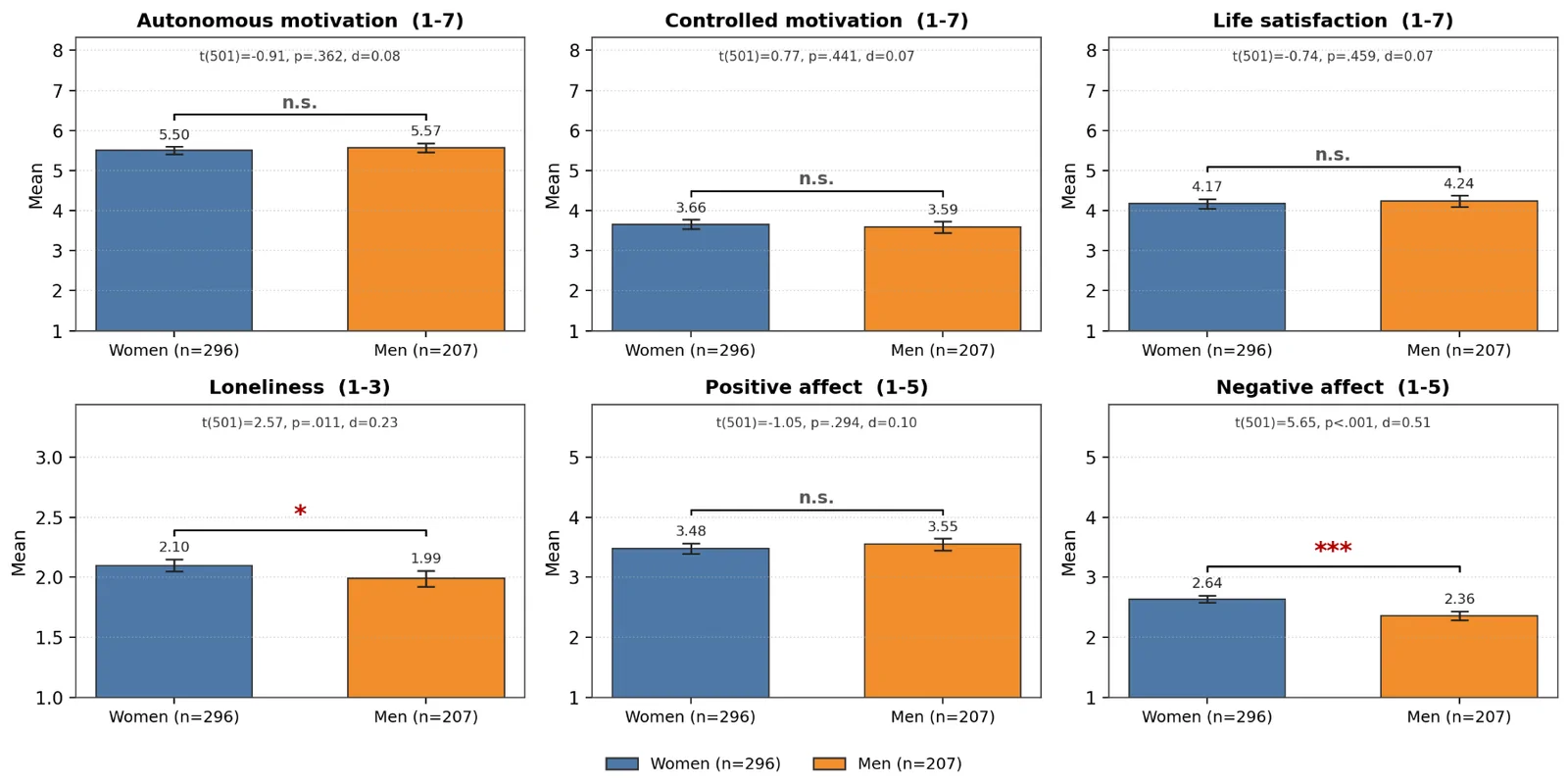
**Mean scores of all six study variables by gender, with error bars (95% confidence interval)**. Asterisks indicate statistically significant differences (*p < 0.05; ***p < 0.001); n.s., not significant.

### By Age Group

ANOVA showed significant differences in autonomous motivation (F[2, 500] = 21.91; *p* < 0.001; $\eta^{2}$*p* = 0.08), controlled motivation (F[2, 500] = 16.11; *p* < 0.001; $\eta^{2}$*p* = 0.06), positive affect (F[2, 500] = 16.68; *p* < 0.001; $\eta^{2}$*p* = 0.06) and loneliness (F[2, 500] = 7.78; *p* < 0.001; $\eta^{2}$*p* = 0.03). Life satisfaction (F[2, 500] = 0.72; *p* = 0.487; $\eta^{2}$*p* = 0.003) and negative affect (F[2, 500] = 1.05; *p* = 0.351; $\eta^{2}$*p* = 0.004) did not differ significantly across age groups. All four significant and both non-significant age contrasts are reported in Table 4, for the first time, displayed jointly in Fig. 4. Bonferroni post hoc tests indicated that the over-50 group showed significantly higher autonomous motivation and positive affect, and lower controlled motivation and loneliness, than the group aged 35 or under (*p* < 0.01 in all cases; Fig. 4).

**Fig. 4.**
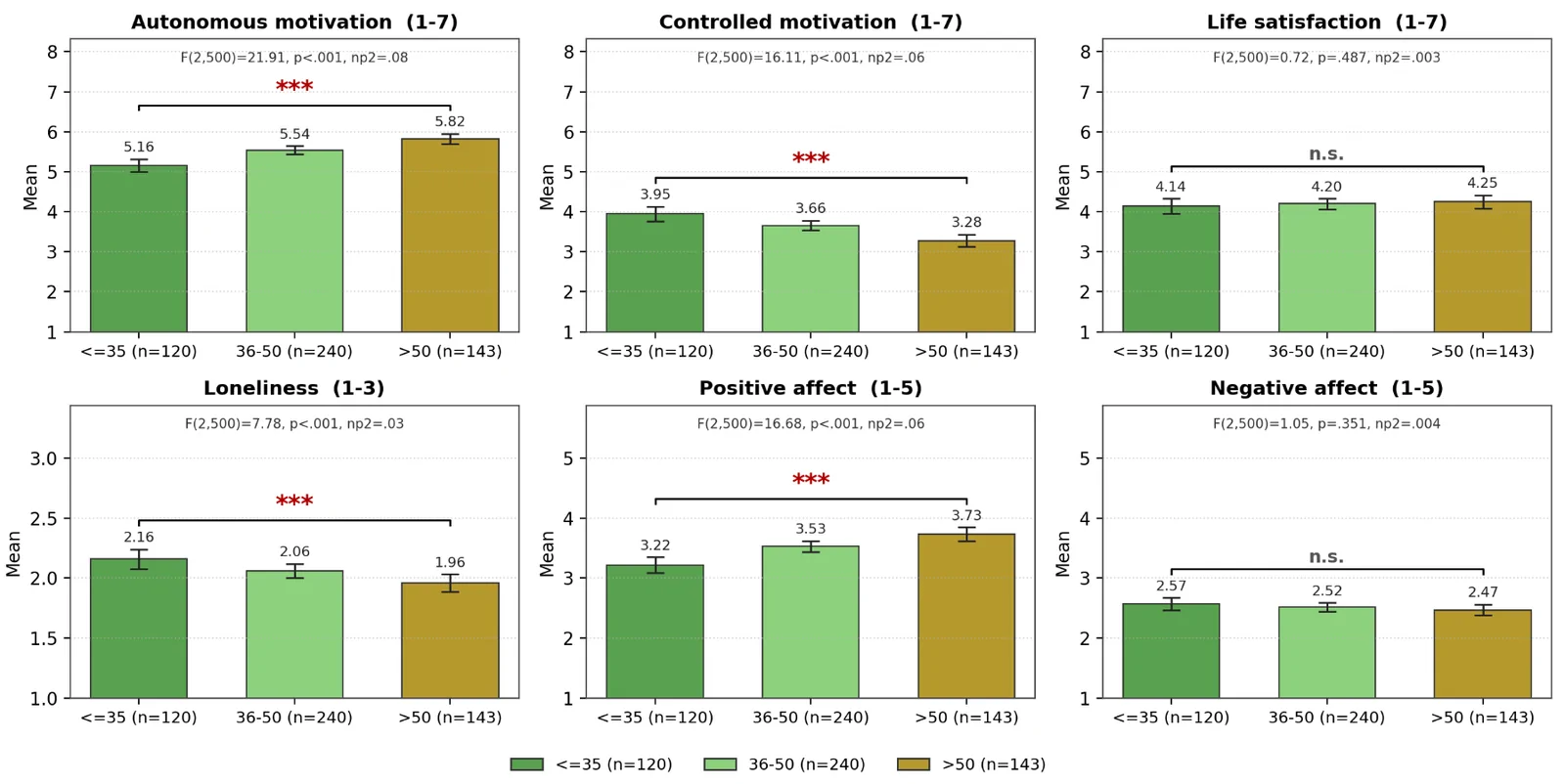
**Mean scores of all six study variables by age group, with error bars (95% CI)**. Significance markers reflect the omnibus ANOVA; Bonferroni post hoc tests locate the difference between the ≤35 and >50 groups. ***p < 0.001. n.s., not significant; ANOVA, analysis of variance.

### By Place of Residence

Teachers living outside large cities, compared with those in large cities, showed significantly higher levels of loneliness (*t*[501] = –6.99; *p* < 0.001; d = –0.62), a moderate-to-large effect. Differences in negative affect were marginal (*t*[501] = –1.84; *p* = 0.066; d = –0.19), and no differences were found in life satisfaction (*t*[501] = 0.97; *p* = 0.331) or positive affect (*t*[501] = 0.93; *p* = 0.354). Autonomous (*t*[501] = 0.53; *p* = 0.594) and controlled motivation (*t*[501] = –0.45; *p* = 0.653) likewise did not differ by residence, so the residence effect was specific to loneliness (Fig. 5).

**Fig. 5.**
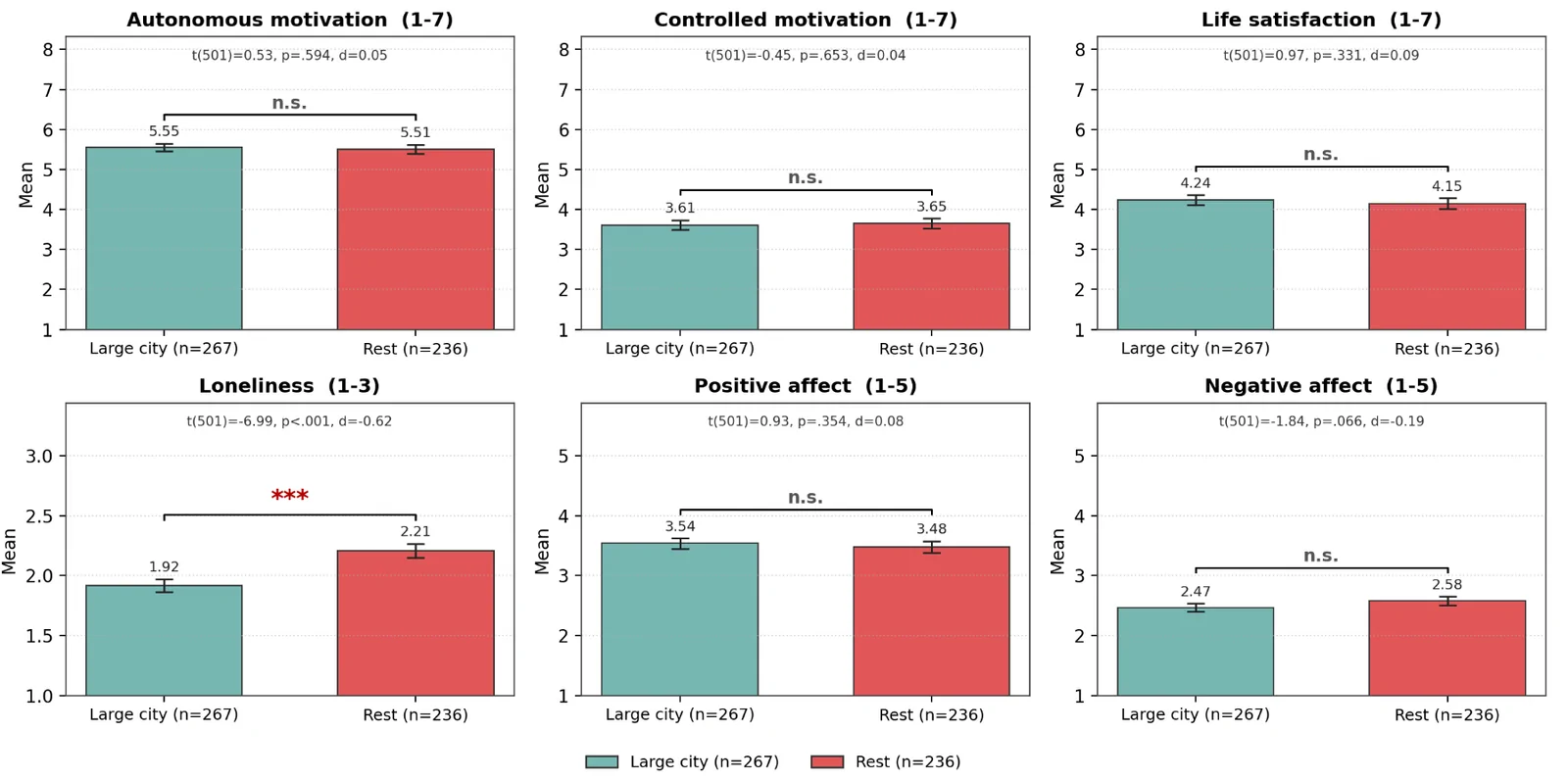
**Mean scores of all six study variables by place of residence, with error bars (95% CI)**. ***p < 0.001. n.s., not significant.

### By Field of Knowledge

ANOVA revealed significant differences in life satisfaction (F[2, 500] = 8.75; *p* < 0.001; $\eta^{2}$*p* = 0.03) and positive affect (F[2, 500] = 5.44; *p* = 0.005; $\eta^{2}$*p* = 0.02). STEM teachers showed the lowest life satisfaction, significantly below that of health-sciences teachers and of those in other fields (*p* < 0.01; Fig. 6). No significant differences by field were found in autonomous motivation (F[2, 500] = 0.32; *p* = 0.727), controlled motivation (F[2, 500] = 0.45; *p* = 0.639), loneliness (F[2, 500] = 0.51; *p* = 0.598) or negative affect (F[2, 500] = 0.68; *p* = 0.510). Although statistically significant, the field effects on life satisfaction ($\eta^{2}$*p* = 0.03) and positive affect ($\eta^{2}$*p* = 0.02) are small according to Cohen’s benchmarks, accounting for roughly 2% to 3% of the variance; they should therefore be read as genuine but modest and interpreted with caution [32].

**Fig. 6.**
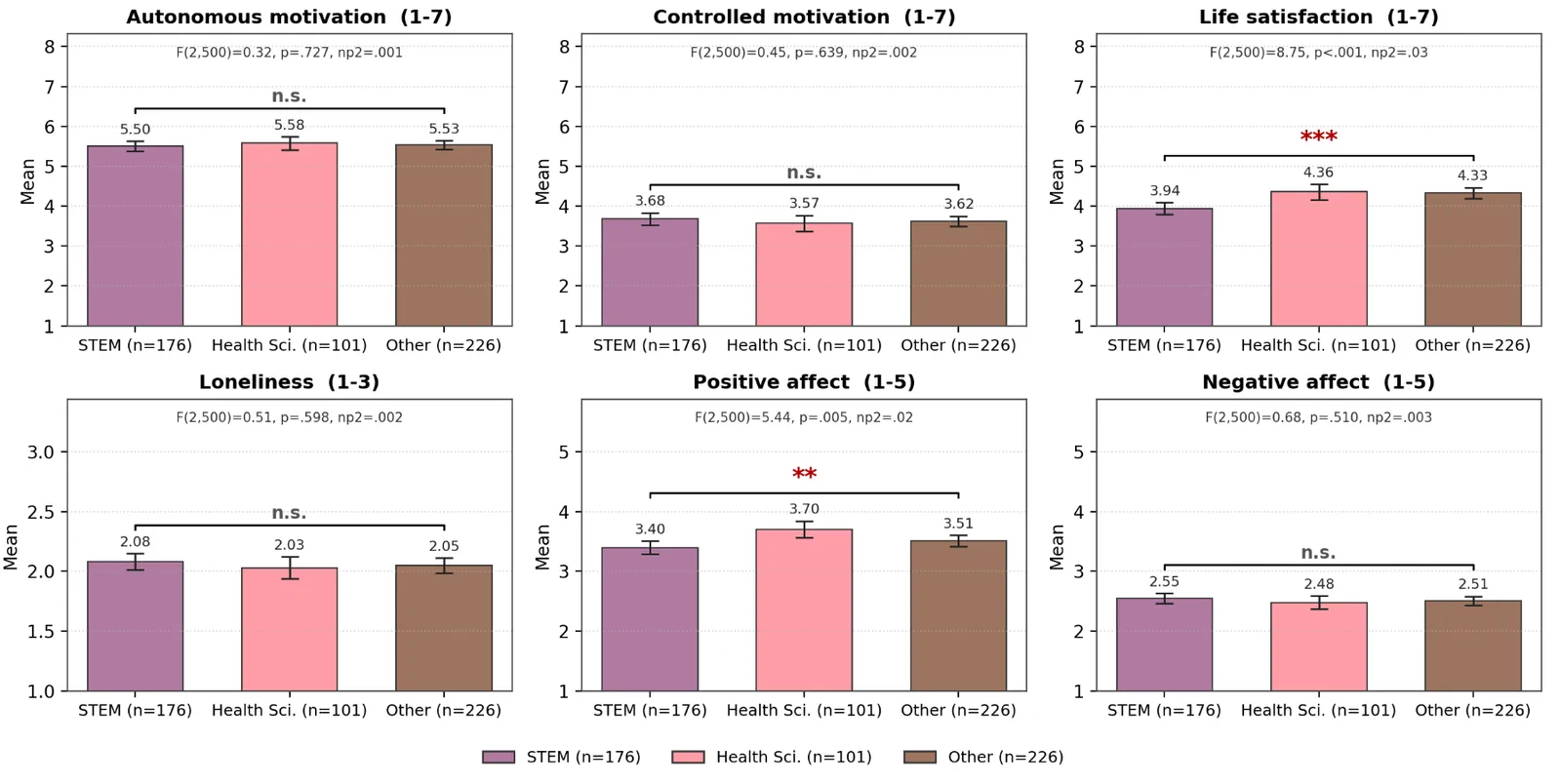
**Mean scores of all six study variables by field of knowledge, with error bars (95% CI)**. Asterisks indicate significant omnibus effects (**p < 0.01; ***p < 0.001). n.s., not significant

All non-significant subgroup contrasts (all *p* > 0.05) are reported numerically in Table 4; the complete set of the six study variables for each subgroup is plotted in Figs. 3–6.

### Cluster Analysis: Motivational Profiles

The k-means analysis identified three clearly differentiated clusters (Table 5 and Fig. 7). The first cluster, termed autonomous-flourishing (n = 160; 31.8%), was characterised by the highest autonomous motivation, the highest life satisfaction and positive affect and, notably, the lowest loneliness and negative affect. The second, ambivalent-intermediate (n = 154; 30.6%), combined relatively high autonomous motivation with the highest controlled motivation and a level of loneliness similar to that of the third group. The third cluster, demotivated-vulnerable (n = 189; 37.6%), showed the lowest autonomous motivation and life satisfaction, the lowest positive affect and high levels of loneliness.

**Table 5.** **Profile of the three clusters: means (SD) on raw scores and ANOVA validation for all six variables**.

Variable	C1 Autonomous-flourishing	C2 Ambivalent	C3 Dmotivated-vulnerable	F(2, 500)	*p*	$\eta^{2}$ *p*
	(n = 160)	(n = 154)	(n = 189)			
Autonomous motivation	6.09 (0.61)	5.85 (0.59)	4.80 (0.63)	221.68	<0.001***	0.47
Controlled motivation	2.92 (0.86)	4.13 (0.82)	3.82 (0.92)	83.33	<0.001***	0.25
Life satisfaction	4.90 (0.94)	4.36 (0.84)	3.48 (0.79)	123.13	<0.001***	0.33
Loneliness	1.66 (0.36)	2.24 (0.35)	2.25 (0.43)	128.79	<0.001***	0.34
Positive affect	3.99 (0.57)	3.84 (0.47)	2.84 (0.50)	260.20	<0.001***	0.51
Negative affect	2.16 (0.51)	2.75 (0.55)	2.65 (0.49)	58.64	<0.001***	0.19

Note: All F(2, 500) contrasts were significant at *p* < 0.001 (*** in the dedicated significance column). $\eta^{2}$*p*, partial eta squared. Bonferroni post hoc tests showed that, for loneliness, C1 < C2 ∼ C3, with C2 and C3 not differing significantly. The association with sociodemographic variables was significant for age (χ^2^[4] = 45.32; *p* < 0.001), gender (χ^2^[2] = 14.51; *p* < 0.001), residence (χ^2^[2] = 15.48; *p* < 0.001) and field (χ^2^[4] = 10.65; *p* = 0.031). ANOVA, analysis of variance; SD, standard deviation.

**Fig. 7.**
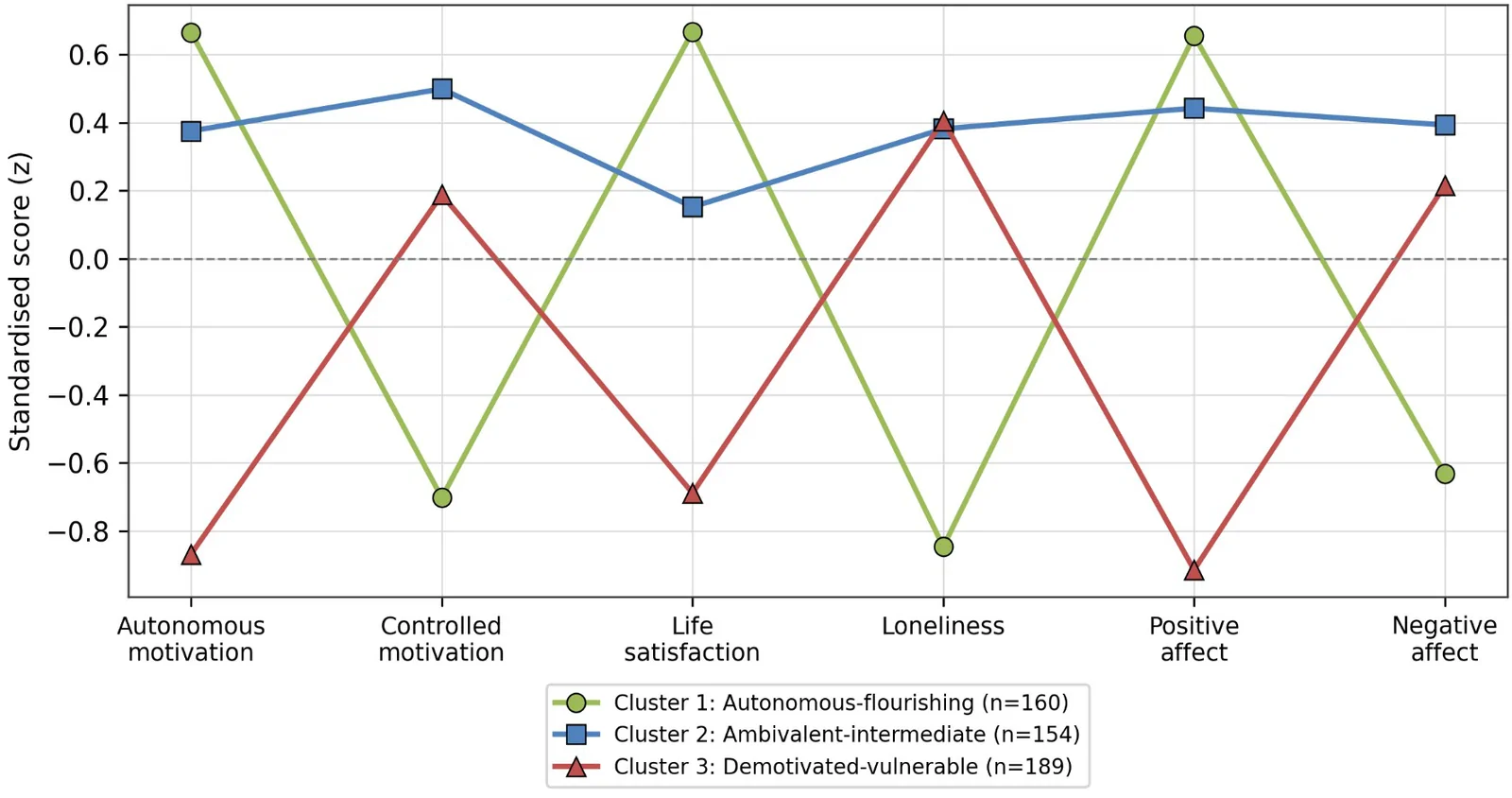
**Motivational and well-being profiles (standardised z scores) by cluster**. The dashed line represents the sample mean (z = 0).

The validation ANOVAs confirmed highly significant differences among the clusters on all six variables (all *p* < 0.001), with large effect sizes, particularly for positive affect ($\eta^{2}$*p* = 0.51) and autonomous motivation ($\eta^{2}$*p* = 0.47). Bonferroni-corrected post hoc comparisons clarified the source of these differences. For loneliness, the autonomous-flourishing profile scored significantly below the other two profiles, whereas the ambivalent-intermediate and demotivated-vulnerable profiles did not differ from each other (2.24 versus 2.25; *p* > 0.05). The large omnibus effect size for loneliness ($\eta^{2}$*p* = 0.34) is therefore driven almost entirely by the markedly lower scores of the first profile, and not by a graded difference across the three groups; this nuance prevents the omnibus statistic from being misread as evidence of three distinct loneliness levels. The most novel result, relative to the prior literature, is that the most motivated profile was simultaneously the least lonely. This pattern differs from that reported under pandemic remote-teaching conditions, in which the profile with the highest intrinsic motivation paradoxically reported greater loneliness [29]. Under conditions of normalised face-to-face activity, autonomous motivation and social connectedness converge coherently.

Because the clusters were derived exclusively from the six psychological variables, their uneven distribution across demographic categories is an expected descriptive consequence of the correlations between those variables and characteristics such as age or gender. It should not be read as evidence that demographic factors structurally determine profile membership; the chi-square associations reported below are descriptive rather than explanatory.

### Regression Analysis

A hierarchical multiple regression model was fitted to predict positive affect (Table 6). In the first step, the sociodemographic variables (age, gender, residence and field) were entered, accounting for 7.5% of the variance (R^2^ = 0.08; F[4, 498] = 10.14; *p* < 0.001). In the second step, the psychological variables (autonomous motivation, controlled motivation and life satisfaction) were added, significantly increasing the explained variance (△R^2^ = 0.39), with the final model reaching 46.7% (R^2^ = 0.47; F[7, 495] = 61.96; *p* < 0.001). Autonomous motivation (β = 0.48; *p* < 0.001) and life satisfaction (β = 0.29; *p* < 0.001) were the most relevant predictors, whereas the sociodemographic variables lost weight once the psychological ones were entered.

**Table 6.** **Hierarchical regression predicting positive affect (N = 503)**.

Step/Predictor		B	SE	β	*t*	*p*	R^2^/△R^2^
Step 1							R^2^ = 0.08
	Age (centred)	0.009	0.003	0.13	2.79	0.005	
	Gender (woman)	0.070	0.065	0.05	1.08	0.281	
	Residence (rest)	0.002	0.065	0.00	0.03	0.973	
	Field (STEM)	–0.128	0.067	–0.08	–1.90	0.058	
Step 2							R^2^ = 0.47; △R^2^ = 0.39
	Age (centred)	0.006	0.003	0.09	2.48	0.014	
	Gender (woman)	0.005	0.049	0.00	0.11	0.915	
	Residence (rest)	0.036	0.049	0.03	0.75	0.452	
	Field (STEM)	–0.064	0.051	–0.04	–1.24	0.217	
	Autonomous motivation	0.418	0.032	0.48	12.89	<0.001	
	Controlled motivation	–0.003	0.026	0.00	–0.11	0.910	
	Life satisfaction	0.204	0.027	0.29	7.66	<0.001	

Note: B, unstandardised coefficient; SE, standard error; β, standardised coefficient; STEM, science, technology, engineering and mathematics. Dependent variable: positive affect.

The unstandardised coefficient for age is small in absolute terms (B = 0.01) because it expresses the change in positive affect associated with each additional year on a 1–5 scale. Across the full age range of the sample (38 years) this corresponds to an increase of approximately 0.34 points, and the standardised coefficient (β = 0.09) confirms a modest but reliable contribution. This continuous result converges with the categorical age analysis reported in Section Descriptive and Reliability Analyses and Table 4, which also located the most adaptive affective profile in the oldest group.

### Mediation Model

The PROCESS analysis (model 4; 5000 bootstrap resamples) indicated that life satisfaction partially mediated the relationship between autonomous motivation and positive affect. Autonomous motivation predicted life satisfaction (a = 0.50; *p* < 0.001), which in turn predicted positive affect (b = 0.21; *p* < 0.001). The total effect (c = 0.54) decreased once the mediator was introduced (c′ = 0.44), and the indirect effect was significant (ab = 0.105; 95% CI [0.073, 0.139]), as the confidence interval did not include zero (Fig. 8). Because the data are cross-sectional, this result represents statistical mediation rather than a demonstrated causal mechanism: the temporal precedence required for a causal interpretation cannot be established here and must be tested in longitudinal or experimental designs [18].

**Fig. 8.**
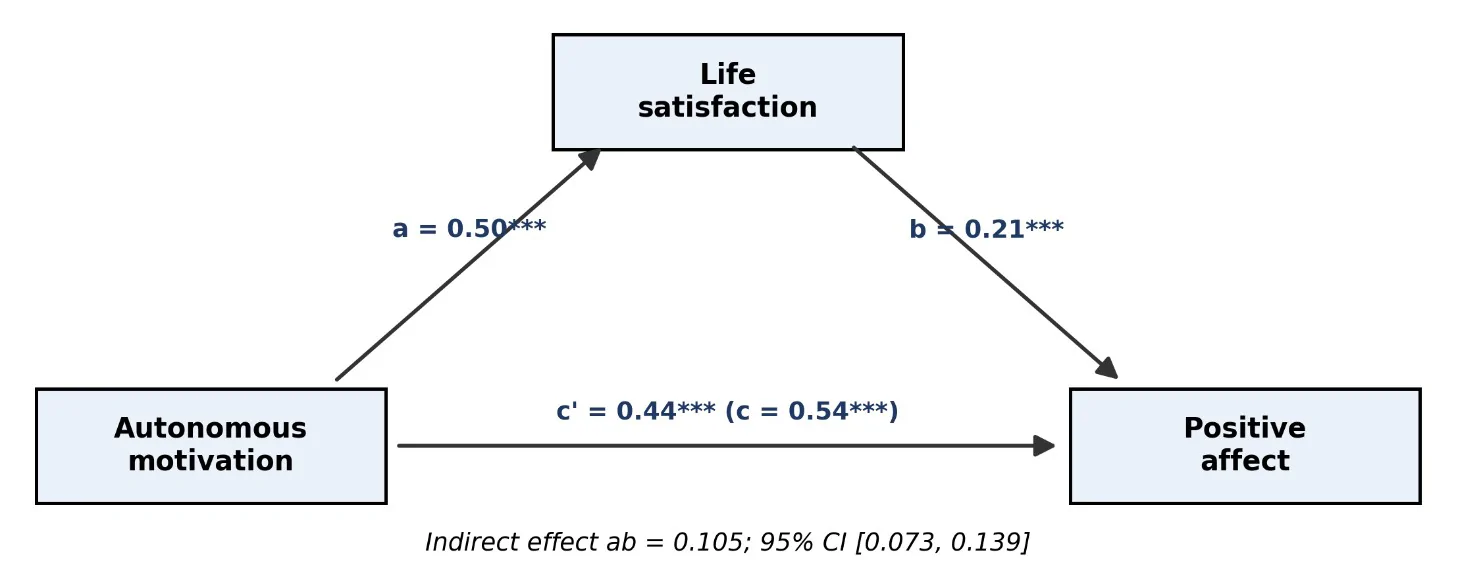
**Mediation model of life satisfaction in the relationship between autonomous motivation and positive affect**. Unstandardised coefficients; ***p < 0.001.

## Discussion

This study analysed the relationships among motivation, life satisfaction, loneliness and affect in Spanish university teachers, identified latent motivational profiles, and examined differences by age, gender, place of residence and field of knowledge. The main finding is the coherent convergence of autonomous motivation, affective well-being and social connectedness, organised around three distinct profiles of vulnerability.

In relation to the first hypothesis, the results confirm the role of autonomous motivation and life satisfaction as statistical predictors of positive affect and as factors associated with lower loneliness. This pattern is consistent with the Self-Determination Theory framework, according to which autonomous regulation of work behaviour fosters positive affective states and greater well-being [3, 5, 8]. The magnitude of the association between autonomous motivation and positive affect (r = 0.62), and its weight in the regression model (β = 0.48), underscore its relevance as a target for intervention.

Regarding gender differences (H2), the findings of higher negative affect and loneliness among women are consistent with the prior literature on the differential distribution of emotional distress [24, 25], and invite consideration of the role of the double burden of paid work and caregiving. The absence of differences in autonomous motivation and life satisfaction qualifies any simplistic reading of female disadvantage, pointing instead to a specific pattern confined to the negative affective pole. This targeted vulnerability converges with earlier findings on sex differences among teachers [28] and with the broader literature linking performance pressure, loneliness and a diminished sense of belonging to burnout symptoms in academic settings [37].

As for age (H3), older teachers displayed a more adaptive profile (higher autonomous motivation and positive affect, lower loneliness), which may reflect processes of professional consolidation, greater contractual stability and the selection of emotionally meaningful goals typical of later career stages [26]. The effect of field of knowledge is particularly interesting: the lower life satisfaction of STEM teachers suggests that disciplinary cultures shape distinct psychosocial environments that may modulate well-being, possibly because of the intensity of productivity pressure and competitiveness in these fields [27]. This reading is coherent with studies identifying academic burnout and impaired social communication as antecedents of reduced life satisfaction across student and professional samples [41, 42, 43]. It must be stressed, however, that these field effects were small in magnitude ($\eta^{2}$*p* = 0.02 to 0.03), so they identify a genuine but limited source of variability rather than a major determinant of well-being.

The most novel finding concerns place of residence. The marked difference in loneliness between teachers in large cities and those elsewhere (d = –0.62) is a contribution that has been little explored in the literature on teacher well-being, and it connects with growing psychiatric concern about the territorial dimension of loneliness and its impact on mental health [19, 20, 21]. This result indicates that well-being promotion policies should incorporate territorial context as a relevant variable, especially within an increasingly hybrid and geographically dispersed university system.

The identification of three motivational profiles (H4) reinforces the usefulness of the person-centred approach [29, 30]. The contrast with the key earlier study, in which the most motivated profile during remote teaching reported greater loneliness [29], represents a relevant theoretical nuance: under face-to-face conditions, autonomous motivation and social relatedness reinforce one another rather than dissociate. Finally, the mediation model (H5) provides evidence that life satisfaction is a statistical pathway through which, in this cross-sectional sample, autonomous motivation translates into greater positive affect, in line with integrative models of subjective well-being.

Several findings were expressed in predictive and mediational terms for statistical clarity. Given the cross-sectional design, these results establish robust associations rather than causal relationships, and the language of prediction should be understood in its statistical sense. The direction of the observed effects is theoretically coherent, but reverse or reciprocal pathways (for instance, positive affect enhancing autonomous motivation) remain plausible and can be disentangled only with longitudinal or experimental evidence [18, 41].

### Implications for Practice

The results point to the value of institutional occupational mental health programmes that support the satisfaction of teachers’ basic psychological needs, pay specific attention to negative affect and loneliness among women and younger teachers, and develop social-connection strategies aimed at teachers living outside major urban centres. Early detection of the demotivated-vulnerable profile can guide selective preventive interventions. Such programmes are especially warranted given that teachers’ emotional exhaustion has been linked to poorer student achievement [42] and that burnout and low job satisfaction are robust predictors of the intention to leave the profession [43]; attending to the affective texture of teaching is therefore a matter not only of staff welfare but also of educational quality [44].

### Limitations

The cross-sectional design precludes causal inferences, despite the use of mediation models. Non-probability sampling limits the generalisability of the results. With respect to generalisability, the convenience and snowball strategy produced a sample that over-represents women and younger teachers relative to the national PDI population (44.6% women and a mean age of 49.5 years in 2023–2024) [33], so the prevalence estimates and subgroup proportions reported here are sample-specific and must not be extrapolated to the whole Spanish academic workforce. The use of self-report measures may introduce social desirability and common-method variance biases. Common-method variance was examined with Harman’s single-factor test, which did not reveal a dominant method factor [38]; nevertheless, exclusive reliance on self-report cannot rule out residual bias, and the incorporation of objective or multi-informant indicators is recommended. Operationalising residence dichotomously simplifies a complex territorial phenomenon. Future research should incorporate longitudinal designs, representative samples and objective measures of social isolation, and should replicate the profile structure in other university systems. Longitudinal evidence of the type provided by recent prospective workplace-loneliness research [18] is specifically needed to confirm the proposed temporal sequence.

## Conclusions

The findings support the role of autonomous motivation and life satisfaction as protective factors for the affective well-being of Spanish university teachers, and reveal relevant differences by gender, age, residence and field of knowledge. The identification of three motivational profiles, and particularly the convergence of high motivation and low loneliness in the most adaptive profile, adds a novel nuance to the existing evidence. The marked territorial difference in loneliness underscores the value of developing occupational mental health promotion programmes that are sensitive to the sociodemographic diversity and territorial context of teaching staff. Although the cross-sectional design calls for longitudinal confirmation, the convergent pattern of results identifies autonomous motivation as a priority target for occupational mental-health promotion.

## Availability of Data and Materials

The data supporting the results of this study are available from the corresponding author upon reasonable request. For data protection reasons, the dataset is not publicly accessible.
